# Repetitive Sprinting and Running Fatigue in Children with Different Levels of Motor Competence

**DOI:** 10.3390/children12020135

**Published:** 2025-01-26

**Authors:** Dané Coetzee, Wilmarié du Plessis, Bouwien Smits-Engelsman

**Affiliations:** 1 Physical Activity, Sport and Recreation (PhASRec), Faculty of Health Sciences, North-West University, Potchefstroom 2531, South Africa; dane.coetzee@nwu.ac.za (D.C.); wilmarie.duplessis@nwu.ac.za (W.d.P.); 2 Department of Health & Rehabilitation Sciences, Faculty of Health Sciences, The University of Cape Town, Cape Town 7925, South Africa

**Keywords:** DCD, running, fatigue, motor competence, CRISP, PERF-FIT

## Abstract

**Background:** Children with motor delays often experience challenges in health-related fitness, but the impact on running skills remains unclear. Previous research has shown that children with motor coordination problems have lower cardiorespiratory fitness, muscle strength, endurance, and higher body weight compared to peers. Few studies have examined anaerobic capacity, muscular power, endurance, running performance, and fatigue in children with developmental coordination disorder (DCD). This study aims to compare repetitive running and running-induced fatigue in typically developing children and those with varying degrees of motor coordination problems. **Methods:** Groups were classified using the Movement Assessment Battery for Children, second edition (MABC-2), as probably having DCD (p-DCD, ≤5th percentile, age 9.7 (SD 1.6), n = 141), at risk for DCD (r-DCD, 6th–16th percentile, age 9.9 (SD1.6), n = 160), and typically developing (TD, >16th percentile, age 9.6 (SD 1.6), n = 191). Anaerobic fitness and fatigue were assessed using the Children’s Repetitive and Intermittent Sprinting Performance test (CRISP), while lower and upper body muscular strength, running, and agility were measured with the performance and fitness (PERF-FIT) test battery Power and Agility subscale. Age groups (6–9 and 10–12 years) were analyzed to determine when performance deficits emerged. **Results:** The p-DCD group was significantly slower, had less power, and fatigued more than the r-DCD and TD children (*p* < 0.01). This was already clearly the case in the 6–9-year-olds, who slowed down already after the first runs, while the older poorly coordinated children started slower than their peers and showed a more gradual decrease in performance over the runs. **Conclusions:** Moderate coordination differences between r-DCD and TD children did not significantly impact fatigue, but p-DCD children exhibited greater fatigue due to overestimating their start speed, higher body weight, lower power, and reduced agility, especially in younger age groups. (Too) High starting speed, especially in the younger less coordinated children (p-DCD), is likely to lead to more fatigue.

## 1. Introduction

Developmental coordination disorder (DCD) is the recommended diagnostic label for children experiencing pronounced coordination challenges that disrupt their abilities to engage in daily activities (e.g., getting dressed, brushing teeth, playing ball games) and academic tasks, which cannot be explained by any intellectual disability or identifiable neurological disease. These children tend to encounter difficulties in various activities, incorporating gross motor activities like running, jumping, throwing, and catching, as well as fine motor activities including handwriting. The results of a recent review indicate that children with DCD have lower physical fitness performance compared to their typically developing (TD) peers [1]. Over the past decade, it has been well-documented that children who struggle with motor skills have lower levels of aerobic and anaerobic fitness [2,3,4,5,6]. The inability to acquire the appropriate motor skills greatly limits the development of a healthy level of physical fitness. They showcase decreased physical activity resulting in lower cardiorespiratory fitness levels, muscle strength, muscular endurance, and a higher body composition than their peers [7,8,9]. This means that the overall fitness levels (strength, anaerobic fitness, and aerobic capacity) need to be examined when an intervention program is developed for children with lower levels of motor skills as limitations in all these areas can be found [1]. Further, our meta-analysis suggests a need for motor assessments that more closely mirror real-life activities (i.e., ecological validity) across different performance domains. Although running is a daily activity for most children, running items, long enough to tap into the anaerobic capacity, are not included in the most frequently used motor performance tests. Most research has focused on investigating aerobic fitness of children with DCD because it is related to the risk for health problems such as cardiovascular disease [10,11,12,13]. However, predominantly anaerobic capacity is needed when running during active play or recreational sports. Yet in young children, only a few studies have focused on anaerobic capacity, which includes power, muscular endurance, and fatigue [10,11,12,13].

### 1.1. Development of Running

Running is a complex skill that is a combination of coordination, motor planning, agility, and strength and is often overlooked as an important skill that also needs to be learned and later trained [14,15,16]. To understand the mechanism of running, one should first look at the three developmental stages of running. Usually, children begin to run around the age of two, and this is long before they partake in any organized physical activities. During the early stages, also known as the initial or discovery stage of running, it will seem like the child’s arm swing appears stiff and arms are swinging out from the body. The leg swing is also very limited, and the legs make uneven strides with no obvious flight phase visible. Lastly, with no flight phase present, there will always be one foot on the ground, and the child will have a wider base of support while running to maintain balance [14,15,16,17,18,19].

The next stage, also known as the intermediate or developing stage, usually occurs when the child is between the ages of three and six years. In this stage, the arm swing starts to move closer to the body and arms are swung further to the front and back. The leg swing improves, leading to an increase in speed and improved stride length. The stance leg also starts to straighten more during the push-off phase to give more momentum. Lastly, a limited flight phase is evident where both feet are off the ground for a brief moment.

The final stage of the development of the running skill is the mature or consolidating stage, which usually occurs between six and nine years. In this stage, children start to run more like adults with their arms that are bent at a 90° angle at the elbows, demonstrating an arm swing that is close to the body in the opposite direction of the leg movement. Furthermore, a larger stride length with a definite flight phase is visible. The supporting leg bends slightly upon contact with the ground and then straightens to propel the body upwards during the push-off phase, which increases the running speed [17,18,19].

Agility, strength, and muscular power are all factors integrated during the development of the running phases. During these phases, the center of mass of the body exhibits a motion comparable to a mass attached to a weightless spring, rebounding off the ground [20,21,22,23] and oscillating around an equilibrium point where the vertical force equals the body weight. Equilibrium point models describe running economy based on spring-like behaviors of the musculoskeletal system. During the first part of the contact phase on the stance leg, the spring–mass system is compressed, and potential elastic energy is stored in the muscle–tendon units of the lower limb to be further released during the second part of contact when the spring expands. This stored energy helps with power generation in the push-off phase to get the body into the next flight phase. A longer flight phase demands greater power generated during the contact phase to accelerate and lift the body appropriately. Learning to exploit these elastic properties will improve running efficiency.

### 1.2. Running with DCD

Running is a fundamental movement skill that is not only important for participating in playground activities but also for development of fitness and overall health [24]. Running should therefore be seen as an integrated part of many physical activities, and if development is delayed, this could impact participation in these activities [17,18,19,25]. The results of the Diamond study [26] suggested that children with DCD run with a slower and less efficient running style compared with TD children. One of the reasons could be that children with DCD struggle with the coordination of the running pattern [27] and the efficient use of strength and power. Even in walking, children with DCD tend to be slower with a shorter stride length with less ankle range of movement than TD children [28,29]. According to research by Cairney [30] and Diamond [26], possible reasons for poor running could be due to slower contraction speed and lower ankle plantar flexor power. This leads to compensatory hip flexor movements at the push-off phase and could also affect the release of the potential elastic energy normally stored in the muscle–tendon system. The reduced running speed in children with DCD could be linked to their ineffectiveness in applying the absorption/generation plantar flexor strategy while running, which in turn could restrict their running speed or induce earlier exertion.

### 1.3. Repetitive Sprinting and Fatigue

The ability to produce a stable running performance over a series of sprints with short recovery is called repetitive sprinting ability [31]. A test to measure repeated sprinting performance and fatigue is the Children’s Repetitive and Intermittent Sprinting Performance test or CRISP. Fatigue can be seen as a continuous, multifaceted process that occurs during high-intensity exercise, for example, while sprinting [32,33]. It includes both central and peripheral mechanisms that temporarily reduce the power-generating capacity of the integrated neuromuscular system. Fatigue also refers to the inability to continue with a given activity or exercise at a given intensity. It is therefore important to understand how children with DCD experience fatigue, if this is different from their TD peers, and if fatiguing during sprinting is different in younger and older children [32,33].

Currently, one study investigated if the CRISP test could induce fatigue among school-aged children (7–12 years) and tested the validity of the test in children with probable DCD (p-DCD) and TD peers [12]. These researchers reported that the CRISP test could be used to induce fatigue among children. It was also reported that children with p-DCD exhibited lower anaerobic capacity, including muscular power and muscular endurance, in comparison to their TD peers. Despite this, fatigue levels were similar between the two groups. However, one of the limitations of this study was that only a small number of children (n = 42) were evaluated, and the study lacked power for the groups to be divided into different motor proficiency groups and age groups.

Several studies have been published, indicating that children with DCD usually have a higher body mass index (BMI) than their peers [34,35,36,37,38], and it is well-known that body weight could also have an impact on a child’s endurance and running skills [39,40,41]. The study conducted by Haapala and associates [41] on children between the ages of six and eight years reported that children who had higher body fat percentages performed poorer in the different running and jumping tests than their peers. A reason could be that during these skills children have to carry their weight, which could compromise musculoskeletal functions, especially in overweight and obese children [39,42].

To conclude, it was indicated that children with DCD tend to produce less optimal movement patterns than their peers, leading to slower running and possibly early exertion. Physical fatigue so far was only reported in one study. Other factors to take into account are the reported lower strength and power and the higher prevalence of being overweight in children with DCD.

Therefore, this study aimed to compare sprinting time, power, and fatigue during repetitive sprinting in 6–12-year-old children with different levels of motor competence and in different age groups. The MABC-2 total score was used to define the three motor coordination groups.

This study had the following three sub-aims.

(1) To confirm that children with lower MABC-2 scores are slower in repetitive sprinting. To test if this lower performance is already present in young children (6–9 years) or only in older ones (10–12 years).
(2) To examine if induced fatigue (decay in performance) during repetitive sprinting (CRISP test) is larger in children who score lower on the classification of the MABC-2. If so, to test if the increase in running time and reduction in power are age group-dependent (6–9 years versus 10–12 years).
(3) To study if BMI, functional strength (power lower and upper extremities), agility (ladder run, ladder step, and side jump), and motor performance (MABC-2) are important explanatory factors for the level of fatigue on the CRISP.

Considering previous studies, it was hypothesized that children with probable DCD (p-DCD), compared to typical developed children (TD), would show a longer running time, lower power, and higher fatigue indexes during running compared to TD. The at-risk-for-DCD (r-DCD) group is expected to show results between the p-DCD and TD.

## 2. Materials and Methods

### 2.1. Study Design

This study was an observational cross-sectional case-controlled study including r-DCD, p-DCD, and TD groups. Data were gathered from children aged 6–12 years, obtained during the years 2019, 2022, and 2023, as part of the PERF-FIT study.

### 2.2. Participant Selection

Children in grades 1–6 of four schools were invited to participate. All children whose parents signed the written informed consent and gave assent themselves were included (n = 500). Children’s motor coordination was measured using the Movement Assessment Battery for Children-2 (MABC-2 test) [43]. Of the 500 children, 191 children scored above the 16th percentile on the MABC-2 test (TD), 162 scored at or below the 16th percentile and above the 5th (at risk for DCD), and 147 children scored at or below the 5th percentile (probable DCD) on the MABC-2 test. Because not all the DSM-5 criteria could be confirmed, the group identified as having severe movement difficulties will be referred to as p-DCD group [44]. After checking the data for outliers and missing values, data of 5 children were taken out (2 were extremely slow on the CRISP, both p-DCD, and 3 children had 1 item that was scored as fail (F); so, no total MABC-2 scores were available). Data were analyzed for 216 children between 6 and 9 years old and 284 children between 10 and 12 years of age (see Table 1).

### 2.3. Measurements

#### 2.3.1. Movement Assessment Battery for Children Test-2 (MABC-2 Test)

The Movement Assessment Battery for Children, second edition (MABC-2), is a test that can be used to identify children between the ages of 3 and 16 years with impaired motor function [43]. The test consists of three age bands: age band one (3 to 6 years), age band two (7 to 10 years), and age band three (11 to 16 years). For this study, only age band two was used. There are eight test items in each age band, with three categories, namely, manual dexterity, aiming and catching, and balance. The raw score was converted to a standard score and percentile. Percentile scores of 5 or less indicate severe motor problems, while a score between 6 and 16 suggests the child is at risk of having movement difficulties. A percentile ranking above 16 indicates performance in the normal range of the tested motor skills. The test is a reliable measuring instrument with a test–retest reliability of 0.88–0.99 for the component scores and 0.97 for the total test scores [45]. Since there were no valid motor tests with norms for African countries, we elected to use the Dutch norms, as was done in our earlier studies involving South African children to make the studies comparable [2,46].

#### 2.3.2. The Children’s Repetitive and Intermittent Sprinting Performance (CRISP) Test

The CRISP test was used to evaluate anaerobic fitness and fatigue in the participants. This assessment comprised of six 30 m sprints at maximum speed, combined with brief recovery periods. Each participant was required to sprint as rapidly as possible from one line to the other, with explicit instructions not to decelerate before crossing the finish line. Sprint times, as taken with stopwatches, indicating the time taken to complete each run, were recorded as a key metric. Before the test, each child underwent a 1 min warm-up, and the test protocol was thoroughly explained and demonstrated, either by the tester or by observing other children undergoing the same test, ensuring a clear understanding of the test requirements. Throughout the testing period, participants received verbal encouragement. To facilitate the comparison between the TD and DCD groups, additional outcomes were derived from the sprint times. Mean power (MP) was used as a measure of anaerobic capacity and was calculated using the sprint time of the six runs and the weight of each participant. Power was calculated by using the following formula: power [(body mass × distance^2^)/sprint time^3^] [47]. Mean and peak power (Watts), which are the average and highest power output of all six sprints, were determined. Greater MP indicates the ability to maintain power output over time and translates into better maintenance of anaerobic performance. Fatigue was measured as a percentage of the difference between the slowest and fastest running times [fatigue index time = (slowest running time − fastest running time/fastest running time) × 100]. Fatigue index power was calculated using the peak and low power data points over the six runs. The children participating in this study did not wear shoes while running because most of them had no suitable footwear.

#### 2.3.3. Performance and Fitness (PERF-FIT) Battery Power and Agility Subscale

The PERF-FIT test battery was employed for this study. The PERF-FIT is a functional measure of motor skill-related fitness for children [48]. The PERF-FIT comprises two subscales: Motor Performance subscale and Power and Agility subscale. The Motor Performance subscale incorporates the motor potential to carry out a physical activity and consists of skill item series for bouncing and catching, throwing and catching, jumping and hopping, and static and dynamic balance. The Power and Agility subscale consists of five test items: running, stepping, side jump (agility and muscular endurance), standing long jump (muscular power of the legs), and overhead throw (muscular power of the arms). The PERF-FIT battery is a valid and reliable test for children aged 5–12 years, with excellent content validity (content validity index ranging from 0.86 to 1.00), good structural validity, excellent inter-rater reliability (ICC. 0.99), and good test–retest reliability (ICC. ≥ 0.80) [48,49,50]. Only the Power and Agility subscale was used in this study.

Agility items:

*Ladder tasks* required the child to run accurately in an agility ladder with specified dimensions (3.5 m + 0.5 m for the turn) [12] and consisted of 2 items (running with 1 step per square and stepping with 2 steps per square).
*The side jump item* involved jumping sideways in the squares of the agility ladder (each square 35 cm by 35 cm) for 15 s. Each participant was instructed to jump as fast as possible from square to square without any mistakes. It measured both muscular endurance and dynamic balance.

Strength items:

*Overhead throw:* Throw item involved throwing a 2 kg sandbag for maximum distance from a kneeling position, as a measure of upper body strength and power.
*Standing long jump:* The standing long jump was used to determine explosive leg power, and the participants should aim to jump as far as possible from the starting position and must land on their feet without falling backward or forward.

All items were performed twice, and the best score (either time or distance) was used for the analysis.

#### 2.3.4. Body Mass Index (BMI)

Height was measured to the nearest 0.1 cm with the use of a portable stadiometer. Body mass was measured to the nearest 0.1 kg with the use of the Omron BF511 scale. Body mass index (BMI) was calculated as mass (kg) divided by height squared (m^2^).

### 2.4. Procedure

The PERF-FIT study, conducted from 2019 to 2023, received approval from the Human Health Research Ethics Committee (HREC; NWU-00491-19-A1) at the North-West University (NWU). Following necessary ethical protocols, permission was granted by the North-West Department of Basic Education to carry out the current investigation within educational institutions. Before commencing data collection for the PERF-FIT study in 2019, 2022, and 2023, meetings were arranged with the principal (gatekeeper) of each school and the principal investigator. The study’s objectives were explained during these meetings, securing participation from the primary schools. Subsequently, a goodwill letter, signed by the principal, affirmed the elementary school’s willingness to participate.

### 2.5. Data Analyses

Data were checked for normality, and appropriate analyses were reported. Data of three children with extreme decay in running time were excluded. Differences in descriptive characteristics among groups were examined using one-way ANOVA for continuous variables and chi-square for categorical variables. To test the effect of the repetitive sprinting, two mixed-model analyses of variance (ANOVA) were applied with the runs (6) as the within-subject factors for time (s) and power (Watts) with motor coordination group (TD, r-DCD, and p-DCD) and age group (2) as between-subject factors. The analysis was further fitted using polynomial contrasts, and post hoc comparison was performed for the motor coordination groups with Bonferroni correction for multiple testing. Univariate analysis of variance was used to examine differences between motor coordination group and age group for the fatigue indexes.

Linear multiple regression models (stepwise) were used to assess the associations between the test items and fatigue indexes. BMI, MABC-total score (SS), agility ladder running (s), agility ladder stepping (s), side jump (#), long jump (cm), overhead throw (cm), and running time in the first run were entered in the regression model as dependent variables, with fatigue index time or fatigue index power as the independent variables. Data analysis was conducted using IBM SPSS (version 29; Armonk, NY, USA).

## 3. Results

### 3.1. Participant Characterictics

**Figure 1:**
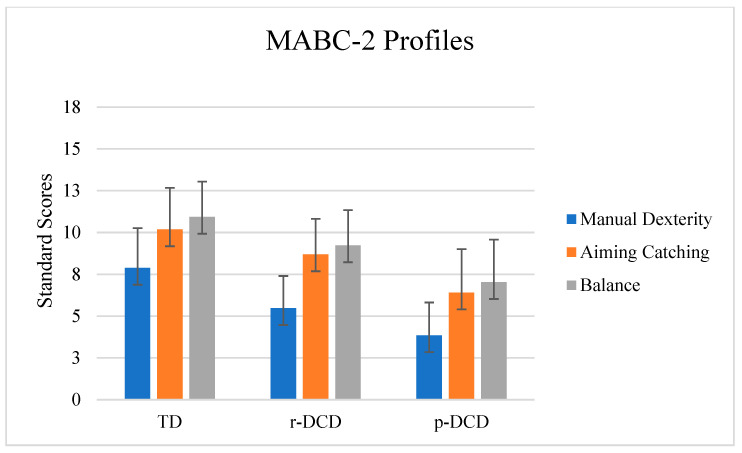
The total group’s MABC-2 standard scores. Manual dexterity was the lowest subscale for the children in all the groups (TD; r-DCD; and p-DCD).

In total, 500 children participated in this study. An overview of the participant characteristics of the TD, r-DCD, and p-DCD groups is shown in Table 1. No statistical differences were found between groups regarding age (*p* = 0.16), weight (*p* = 0.059), and height (*p* = 0.47). Yet, BMI was different (F 5.155 (2496), *p* = 0.006). The p-DCD group had a higher BMI (M = 17.5 ± 3.7) than the TD (M = 16.6 ± 2.7) group (*p* = 0.005). MABC-2 profiles are depicted in Figure 1.

### 3.2. Comparison Between Groups on the Sprint Times of the CRISP Test

To examine differences in the CRISP sprint times between age groups and motor coordination groups, a mixed-model analysis of variance was conducted. Results indicated a significant main effect of age group, motor coordination group, runs, and interactions between running time and age group, motor coordination group, and their combined effects (Table 2).

**Figure 2:**
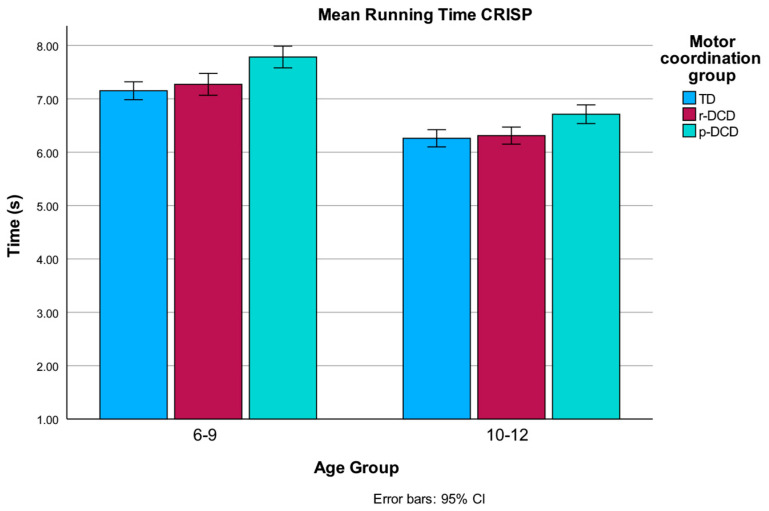
Main effect for age group and motor coordination group on running time.

The 6–9-year-old group was significantly slower over the six runs compared to the 10–12-year-old group. Post hoc analysis showed that the p-DCD group exhibited a longer time to complete the runs compared to both the TD (*p* < 0.001) and r-DCD (*p* < 0.001) groups. The TD and r-DCD groups were not different regarding the mean running times (Figure 2).

**Figure 3:**
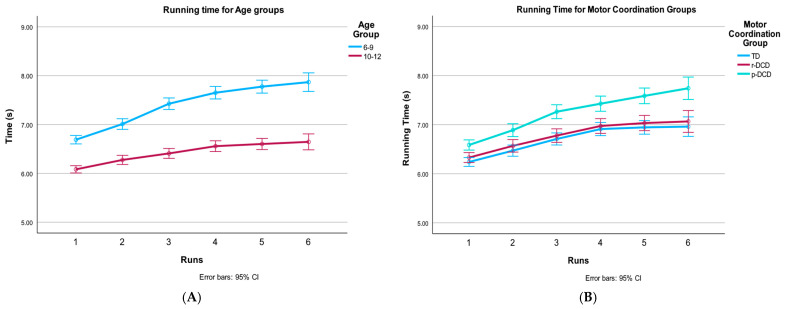

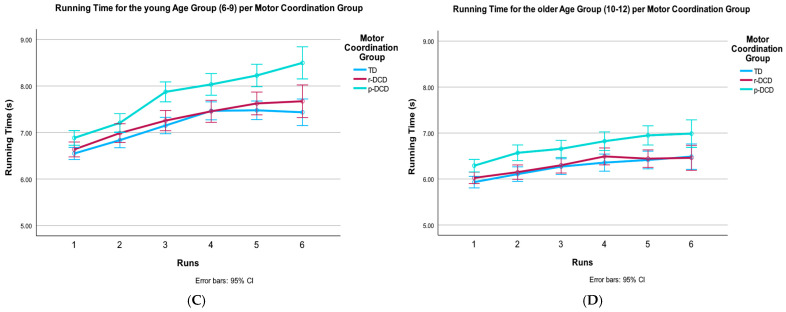
Changes over runs in the different age and motor coordination groups for running time. (**A**) CRISP mean time for runs in the different age groups. Older children are faster. Note differences get larger in the later runs, which explains the runs by age group interaction. (**B**) CRISP mean time for the six runs in the different motor groups. Note differences are large between the two DCD groups, which get larger in the later runs, which explains the runs by motor coordination group interaction, (**C**) Mean time for runs in the 6–9-year-old age group according to the different motor groups. Note the p-DCD group slows down more after the second run, which increases the difference with the other two groups, (**D**) Mean time for runs in the 10–12-year-old age group according to the different motor groups. Note that older children in all groups slow down almost linearly over the six runs. The fact that older children show a comparable slowdown while in the younger children, the p-DCD fatigue more explains the runs by age group by motor coordination group interaction.

Further polynomial analysis of the interaction effects revealed that this effect of changes in running time was quadratic, indicating that overall children’s running times decreased more in the first runs and the curve flattened towards the last runs. These polynomial findings are visually represented in Figure 3A–D. Panel 3A depicts the overall age group difference but also shows that the younger children fatigue more towards the end. In panel 3B, the difference can be seen between the p-DCD group and the two other groups. The latter two groups showed very similar patterns of increased running times. Panel 3C depicts that the p-DCD group slowed down more after the second run in the younger age group, which increases the difference with the other two groups towards the end. Lastly, in panel 3D, all three motor coordination groups of the older children slow down almost linearly over the six runs but with a clear difference between p-DCD and the other two groups. Table 3 presents the CRISP sprint outcomes per age group and motor coordination group.

### 3.3. Comparison Between Groups on the Power of the CRISP Test

**Figure 4:**
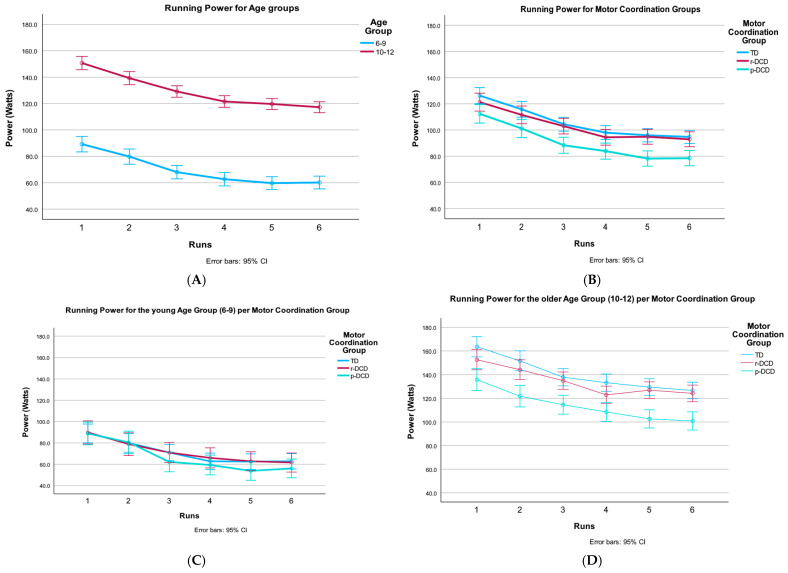
Changes over runs in the different age and motor coordination groups for the running time. (**A**) Power per age group for the CRISP runs. Note the large differences in power, but slowdown has a similar pattern. (**B**) Power per motor coordination group for the CRISP runs. p-DCD has less power over all runs, but slowdown has a similar pattern. (**C**) Power per motor coordination group for the CRISP runs in the 6–9-year-old group. In young children, the difference between motor coordination groups is not significant. Yet, the p-DCD group loses more power after the first two runs, causing the interaction effect. (**D**) Power per motor coordination group for the CRISP runs in the 10–12-year-old group. p-DCD has less power over all runs, which is dissimilar from the pattern in the younger age group.

Results of the mixed-model ANOVA of the CRISP power are presented in Table 4 and visualized in Figure 4A–D. Young children produce less power compared to the older group (4A), and p-DCD children have less power compared to the TD and r-DCD groups (4B). Post hoc analysis revealed the p-DCD group showed lower power compared to TD (*p* < 0.001) and r-DCD (*p* = 0.039) groups; TD and r-DCD were not different. Importantly, the three-way interaction effect reached significance. In the older group (10–12 years old), mean power differed significantly among groups, with the p-DCD group showing lower values compared to the TD (*p* < 0.001) and r-DCD (*p* < 0.001) groups (4D). This was not the case in the younger age group (4C).

### 3.4. Comparison of Fatigue Indexes Between Groups

Table 5 summarizes the statistics for the fatigue index time and fatigue index power on the CRISP test. Comparing across age groups, significant differences in fatigue index time and fatigue index power were observed

**Figure 5:**
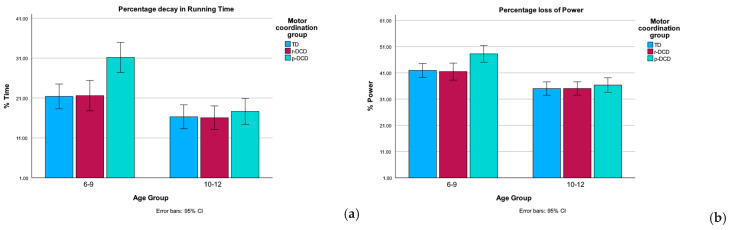
Fatigue index time and fatigue index power on the CRISP test for the different age groups classified into the different motor coordination groups. (**a**) Fatigue index time in the different age groups and different motor coordination groups; (**b**) fatigue index power in the different age groups and different motor coordination groups.

The older age group (10–12 years old) fatigued less compared to the younger group (6–9 years old) (see Figure 5). Also, significant differences in the two indexes among the three motor coordination groups were found. Children with p-DCD fatigued the most.

### 3.5. Associations Between BMI, Agility, Strength, Motor Proficiency, and Fatigue Indexes

In total, five regression models were run to determine the predictive validity of the MABC-2 total standard score, the five PERF-FIT Power and Agility items (ladder run, ladder step, side jumps in 15 sec, long jump, and overhead throw), BMI, and CRISP time in the first run. The last models are shown in Table 6 (fatigue time index) and Table 7 (fatigue power index).

The explained variances by the regression models were 21% for the fatigue time index and 18% for the fatigue power index. Factors significantly predicting the slow down in time (fatigue index time) were long jump distance, BMI, overhead throw distance, the number of side jumps in 15 s, and the starting speed in the first CRISP run. The two ladder items and the total MABC-2 score were not significant predictors for the fatigue index time (Table 6).

Factors significantly predicting the loss in power (fatigue index power) were long jump distance, the number of side jumps in 15 s, the overhead throw distance of the sandbag, and the time needed for the first CRISP run (s) (Table 7). The two ladder items, BMI, and the total movement ABC score were not significant predictors for the fatigue index power.

## 4. Discussion

This study aimed to explore the relationships between motor proficiency, physical fitness, and running fatigue in children aged 6 to 12 years. Specifically, it was examined whether children with lower MABC-2 scores have slower repetitive sprinting performance. Next, fatigue in these children was tested, and lastly, the influence of factors such as BMI, functional strength, agility, and motor performance on fatigue levels was explored.

### 4.1. Motor Proficiency and Sprinting Performance

The present findings confirm that children with poor motor proficiency, particularly those with p-DCD, exhibit lower running speeds and reduced power output during repetitive sprinting compared to TD peers and those at risk for r-DCD. These results were expected based on prior research, which has consistently shown that children with p-DCD struggle with motor planning, interlimb coordination, and the efficient use of strength during dynamic tasks [8,26,27]. The early manifestation of these deficits in younger children (6–9 years) aligns with developmental theories suggesting that motor coordination challenges emerge during early childhood. However, as the gap between p-DCD and TD children becomes more consistent in older children (10–12 years), it suggests that the discrepancies in motor competence may stabilize over time. This adaptation could reflect increased self-awareness or the development of compensatory strategies that allow for more predictable but less efficient movement patterns. Previous research supports the notion that children with DCD often demonstrate lower interlimb coordination and higher variability in movement patterns during demanding tasks, especially running [8,26,27]. Future studies could explore whether targeted interventions at earlier stages can mitigate these disparities.

### 4.2. Age-Related Differences in Fatigue

Younger children (6–9 years) in this study experienced greater fatigue during the repetitive sprinting tasks compared to older children (10–12 years). Additionally, it was also reported that younger children with p-DCD experienced even more rapid fatigue during sprinting tasks compared to their TD peers, despite starting at similar speed and power levels. This outcome aligns with expectations, as younger children typically have less developed anaerobic capacity and energy management skills [51]. The anaerobic capacity for both genders increases with age but is not fully developed until 20 years. This is due to less muscle mass and less glycogen storage at earlier ages [52]. However, the pronounced fatigue in younger children with p-DCD was not entirely anticipated. This rapid performance decline could be attributed to their overestimation of start speed and inefficient movement strategies, which lead to premature energy depletion. The finding that older children with p-DCD exhibited a more stable but consistently reduced performance might reflect their learning to pace themselves, avoiding early exhaustion. The developmental differences in energy systems, combined with the unique challenges faced by children with p-DCD, highlight the need for age-specific interventions focusing on energy management and pacing strategies [51,52].

### 4.3. Running Patterns and Mechanical Factors

Leg length and growth phases play critical roles in running performance, particularly during adolescence, when children experience “adolescent awkwardness”. This phase can temporarily impair coordination, affecting running cadence and contact time [53,54]. Additionally, this study aligns with findings that children with p-DCD have difficulty utilizing the plantar flexor strategy effectively during running, leading to slower speeds and earlier fatigue. This finding was consistent with previous research emphasizing the biomechanical inefficiencies in this population. The inability to store and release elastic energy in the muscle–tendon system during the push-off phase impairs their running economy. This can affect running cadence and contact time [26,30,53,54]. While these results were expected, the specific impact of this inefficiency on repetitive sprinting performance in younger versus older age groups adds a new layer of understanding. Younger children’s reliance on inefficient strategies may exacerbate fatigue; whereas, older children’s compensatory mechanisms might stabilize their performance at a lower level. Interventions targeting the development of effective push-off mechanics could significantly improve running performance.

### 4.4. Power Output and Muscular Endurance

As hypothesized, young children produce less power compared to the older group. This was especially evident in the younger age group, highlighting the interplay between neuromuscular development and motor deficits. The results suggest that these children may have a diminished ability to recruit muscle fibers efficiently during high-intensity tasks, further compounding their performance challenges [12]. Furthermore, the trade-off between accuracy and speed in motor tasks may hinder their performance, especially in fast-changing contexts [55]. Interestingly, while the power output gap between p-DCD and TD children remains significant in the older group, the pattern of power decline is less pronounced, possibly indicating a plateau in the development of muscular endurance. This reinforces the need for targeted functional strength and power training, combined with running technique for this population.

### 4.5. Predictors of Fatigue

The regression analysis identified BMI, agility (as measured by the side jumps), and explosive power in both the upper (overhead throw) and lower (long jump) extremities as significant predictors of fatigue during sprinting. These results were expected, as higher BMI is well-documented to negatively impact endurance and running performance due to the increased physical demand of moving a heavier body [39,40,41,42]. However, while the findings indicated that BMI correlated with overall fatigue measured in time, it did not significantly impact fatigue-related power loss, indicating that neuromuscular coordination and the ability to sustain power over time, rather than body weight alone, is a more critical factor for children with p-DCD. Still, the increased body mass, particularly due to fat accumulation, can impair musculoskeletal function and lead to greater difficulty in endurance activities and running [39,40,41]. In this regard, Haapala and associates [41] reported that higher body fat percentages correlated with poorer performance in running and jumping tasks among children aged six to eight years, and this is likely due to the additional strain on the musculoskeletal system [39,42]. Moreover, researchers have also indicated that when children grow and body mass increases due to more muscle mass, it could lead to greater step lengths while running, which in turn results in better force production, helping with a better power output [53,54]. However, the opposite is also true that when the increase in body mass is due to higher levels of body fat, it could have a negative impact on the power output while running [53,54,56,57]. These biomechanical adaptations made by children with higher BMIs not only alter their running gait but may also predispose overweight children to musculoskeletal injuries and exercise-related discomfort [39,42,58,59]. Thus, while body mass significantly influences running gait, the effects differ markedly depending on body composition. These findings highlight the complex interplay between body composition and physical performance, where increased body fat can impair endurance activities [39,40] and suggest that interventions should prioritize improving agility and power, alongside addressing body composition, to optimize performance and reduce fatigue in children with p-DCD.

### 4.6. Implications for Future Research and Interventions

A notable finding of this study is the lack of significant differences in sprinting performance between r-DCD children and their TD peers, raising concerns about the sensitivity of the MABC-2 classification system in certain populations. This suggests that r-DCD children may perform adequately in gross motor tasks like running but struggle with more complex motor skills requiring fine coordination.

Future research should explore the developmental trajectories of motor deficits in children with DCD, particularly regarding locomotor activities. Longitudinal studies and motor pattern assessments could provide valuable insights into how motor skills evolve and how children navigate challenges related to fatigue and performance.

Intervention programs for younger children should focus on enhancing energy management strategies and running techniques, while programs for older children should emphasize power generation, muscular endurance, and dynamic postural control. Tailored physical activity programs using game-like activities could address the specific needs of children with p-DCD, promoting improved motor skills and overall fitness.

### 4.7. Practical, Scientific, and Theoretical Applications

Practically, the findings highlight the need for early and tailored intervention programs in clinical and educational settings to address motor deficits, enhance physical fitness, and improve energy management strategies in children with p-DCD. Scientifically, this study contributes to the understanding of the interplay between motor coordination, physical performance, and fatigue, emphasizing the importance of anaerobic fitness in functional activities. Theoretically, the results support and extend existing developmental frameworks, providing evidence that motor skill deficits manifest early and can influence physical performance trajectories over time. This underscores the necessity of addressing these challenges during critical developmental periods.

### 4.8. Limitations of the Study

While the results largely align with the existing literature, some unexpected findings, such as the pronounced fatigue in younger children with p-DCD, warrant further investigation. The lack of significant differences in sprinting performance between r-DCD and TD children raises questions about the sensitivity of the MABC-2 classification system in distinguishing motor challenges in this group. Not all of the DSM-5 criteria could be confirmed in the DCD participants in this study, which may limit the external validity of the findings. Future studies should consider using more ecologically valid motor assessments to capture the complexities of real-world performance. Longitudinal research is also needed to track the developmental trajectories of motor deficits and evaluate the long-term impact of early interventions.

## 5. Conclusions

In conclusion, this study enhances the understanding of the relationship between motor coordination, physical performance, and fatigue in typically developing children and children with DCD. Furthermore, the findings of this study have significant implications for pediatric and rehabilitation practices. The observed differences in running speed, power generation, and fatigue levels underscore the need for targeted interventions that address both physical and neuromuscular aspects of motor proficiency. Pediatricians and rehabilitation specialists can use these insights to develop tailored physical activity and therapeutic programs that emphasize improving motor coordination, muscle strength, and endurance. Early intervention strategies focusing on energy management and biomechanical efficiency during locomotor activities, such as running, can prevent further motor skill deterioration. Furthermore, integrating game-like activities into therapy sessions can enhance engagement and promote sustainable improvements in motor performance, fostering better participation in physical and social activities critical for overall child development.

## Figures and Tables

**Table 1:** Characteristics of participants.

Variables	TD Mean (n = 191)	SD	r-DCD Mean (n = 162)	SD	p-DCD Mean (n = 146)	SD
Age (years)	9.6	1.6	9.9	1.6	9.7	1.6
Weight (kg)	31.4	8.5	32.4	7.6	33.6	10.1
Height (cm)	136.5	11.6	137.9	10.7	137.3	11.2
BMI (kg m^−2^)	16.6	2.7	16.9	2.5	17.6 *	3.7
MABC-2 (TSS)	9.8	1.7	6.4 **	0.4	3.9 **	1.2

BMI: Body mass index, MABC-2 test: Movement Assessment Battery for Children test, second edition, TSS: total standard score, SD: standard deviation, TD: typically developing children, r-DCD: at risk for developmental coordination disorder, and DCD: developmental coordination disorder. * Statistically significant between TD and p-DCD groups at *p* < 0.01. ** Statistically significant between TD and r-DCD groups and p-DCD and r-DCD at *p* < 0.01.

**Table 2:** Repeated-measure ANOVA for running time for the CRISP test.

Variable	df	F-Value	*p*-Value
Age Group	1.489	175.48	<0.001
Motor Coordination Group	2.488	17.747	<0.001
Age Group by Motor Coordination Group	2.488	0.197	0.821
Runs	5.485	150.388	<0.001
Runs (Quadratic Effect)	1.489	665.619	<0.001
Runs by Age Group	5.485	19.367	<0.001
Runs by Motor Coordination Group	10.480	2.153	0.019
Runs by Age Group by Motor Coordination Group	10.480	3.383	<0.001

df—Degrees of freedom.

**Table 3:** Mean (SD) sprint time on the CRISP test for TD, r-DCD, and p-DCD groups and statistics.

CRISP Test	TD (n = 191)		r-DCD (n = 162)		DCD (n = 146)		Statistics Motor Coordination Groups		6–9 Years Old (n = 215)		10–12 Years Old (n = 284)		Statistics Age Groups	
	Mean	SD	Mean	SD	Mean	SD	F-Value	*p*-Value	Mean	SD	Mean	SD	F-Value	*p*-Value
Mean Run Time (s)	6.69 *	0.84	6.67 ^#^	0.89	7.13 *^#^	1.00	13.614	<0.001	7.41	1.08	6.41	0.61	41.407	<0.001
Time 1 (s)	6.24 *	0.71	6.25 ^#^	0.70	6.53 *^#^	0.68	11.547	<0.001	6.71	0.74	6.07	0.59	11.547	<0.001
Time 2 (s)	6.46 *	0.81	6.46 ^#^	0.84	6.83 *^#^	0.98	9.194	<0.001	7.01	0.96	6.26	0.68	9.194	<0.001
Time 3 (s)	6.71 *	0.91	6.66 ^#^	0.93	7.15 *^#^	1.14	12.038	<0.001	7.43	1.15	6.40	0.64	12.038	<0.001
Time 4 (s)	6.91 *	0.74	6.85 ^#^	0.97	7.32 *^#^	1.24	8.733	<0.001	7.65	1.23	6.54	0.70	8.733	<0.001
Time 5 (s)	6.95 *	1.03	6.89 ^#^	1.13	7.47 *^#^	1.20	13.089	<0.001	7.78	1.27	6.69	0.77	13.089	<0.001
Time 6 (s)	6.96 *	0.98	6.92 ^#^	1.14	7.48 *^#^	1.33	10.566	<0.001	7.84	1.97	6.62	0.75	10.566	<0.001
Best Run (s)	6.11 *	0.69	6.11 ^#^	0.71	6.20 *^#^	0.70	11.617	<0.001	6.59	0.73	5.93	0.56	17.151	<0.001
Mean Power (Watts)	104.8 *	48.9	110.4 ^#^	46.6	94.09 *^#^	41.14	5.450	0.005	68.88	24.95	130.10	42.04	377.86	<0.001
Fatigue Index Time	−18.75 *	9.31	−18.26 ^#^	9.95	−23.24 *^#^	24.88	5.128	0.006	−24.19	21.01	−16.60	8.09	15.816	<0.001
Fatigue Index Power	38.36	12.52	37.50 ^#^	12.38	41.44 ^#^	15.81	3.568	0.034	43.69	14.37	35.39	11.69	6.998	0.008

SD: Standard deviation, TD: typically developing children, r-DCD: at risk for developmental coordination disorder, p-DCD: probable developmental coordination disorder, * statistical differences between TD and p-DCD, and ^#^ statistical differences between r-DCD and p-DCD.

**Table 4:** Repeated-measure ANOVA for the mean power output of the CRISP runs.

Variable	df	F-Value	*p*-Value
Age Group	1.489	348.694	<0.001
Motor Coordination Group	2.488	8.123	<0.001
Age Group by Motor Coordination Group	2.488	3.937	0.020
Runs	5.485	143.698	<0.001
Runs (Quadratic Effect)	1.489	140.569	<0.001
Runs by Age Group	5.485	0.865	0.505
Runs by Motor Coordination Group	10.480	0.890	0.542
Runs by Age Group by Motor Coordination Group	10.480	2.519	0.005

df—Degrees of freedom.

**Table 5:** Univariate analysis of variance for the fatigue index time and fatigue index power.

Variable	df	F-Value	*p*-Value
** Fatigue Index Time **			
Age Group	1.494	42.64	<0.001
Motor Coordination Group	2.493	5.57	0.004
Age Group by Motor Coordination Group	2.493	1.99	0.138
** Fatigue Index Power**			
Age Group	1.494	47.89	<0.001
Motor Coordination Group	2.493	4.11	0.017
Age Group by Motor Coordination Group	2.493	1.32	0.268

df—Degrees of freedom.

**Table 6:** Regression models predicting fatigue index time from MABC-2 total score, the five PERF-FIT Power and Agility items (ladder run, ladder step, side jumps in 15 sec, long jump, and overhead throw), BMI, and CRISP running time (s) in the first run.

	*B*	SE	BS	t-Value	r^2^	*p*-Value
**Model 5**						
Constant	49.140	7.988		6.152	0.21	<0.001
Long jump	−0.089	0.028	−0.183	−3.213		0.001
BMI	1.325	0.195	0.336	6.809		<0.001
Side jump	−0.362	0.083	−0.200	−4.344		<0.001
Overhead throw	−0.043	0.011	−0.222	−3.927		<0.001
CRISP time first run	−3.461	0.913	−0.209	−3.792		<0.001

*B*: Unstandardized beta, SE: coefficients standard error, BS: standardized beta, and r^2^: r square.

**Table 7:** Regression models predicting fatigue index power from MABC-2 total score, the five PERF-FIT Power and Agility items (ladder run, ladder step, side jumps in 15 sec, long jump, and overhead throw), BMI, and CRISP running time (s) in the first run.

	*B*	SE	BS	t-Value	r^2^	*p*-Value
**Model 5**						
Constant	74.23	9.173		8.103	0.18	<0.001
Long jump	−0.101	0.032	−0.184	−3.183		<0.002
Side jump	−0.435	0.096	−0.213	−4.548		<0.001
BMI	1.148	0.224	0.258	5.137		<0.001
Overhand throw	−0.043	0.013	−0.196	−3.403		<0.001
CRISP time first run	−3.385	1.048	−0.181	−3.230		0.001

*B*: Unstandardized beta, SE: coefficients standard error, BS: standardized beta, and r^2^: r square.

## Data Availability

The data of this study are available upon request from the corresponding author.
